# Advances in the application of assisted reproductive technology in fertility preservation for female patients with malignant tumours of the reproductive systems

**DOI:** 10.1080/07853890.2026.2650869

**Published:** 2026-04-15

**Authors:** Qian Sun, Yu Su, Xiaoju Zhu

**Affiliations:** a Department of Reproductive Center, Daping Hospital (PLA Army Characteristic Medical Center), Army Medical University (Third Military Medical University), Chongqing, China; b Department of Oncology, Daping Hospital (PLA Army Characteristic Medical Center), Army Medical University (Third Military Medical University), Chongqing, China

**Keywords:** Assisted reproductive technology, female reproductive system, malignant tumours, fertility preservation, oncofertility

## Abstract

**Background:**

Rising female reproductive system malignant tumours and gonadotoxic treatments increase infertility risks in reproductive-age women, many of whom lack sufficient fertility preservation (FP) information.

**Objective:**

To review advances, status, challenges and ethical/legal aspects of assisted reproductive technology (ART) for FP in these patients.

**Methods:**

This narrative review synthesized literature from PubMed, Embase, Web of Science and Cochrane Library (keywords included ART, FP, reproductive system malignancies; literature deadline: January 2022), incorporating original studies, reviews, meta-analyses and guidelines from ASRM, ESHRE, ASCO.

**Results:**

ART has advanced significantly in FP but faces gaps in protocol standardization, accessibility, clinical translation and psychosocial impacts, due to tumour/treatment heterogeneity and psychological factors. Multidisciplinary collaboration, provider training and evidence-based guidelines are needed; future research should focus on ovarian tissue transplantation safety, low-cost technologies, clinical translation of novel approaches, global protocols and ethical frameworks.

**Conclusion:**

ART facilitates FP, but standardized guidelines, multidisciplinary collaboration and long-term outcome research are essential to optimize care and patient autonomy.

The increasing prevalence of malignant tumours of the female reproductive system poses significant challenges to women’s health, particularly regarding fertility preservation (FP). As the incidence of cancers such as ovarian, endometrial and cervical cancer rises, the need for effective strategies to maintain reproductive function in affected women becomes increasingly urgent. Current projections indicate that the number of women diagnosed with reproductive cancers will continue to grow, driven primarily by factors such as advanced maternal age at first childbirth and lifestyle changes. These trends highlight the critical intersection of oncology and reproductive medicine, as many women diagnosed with malignancies are of reproductive age and may wish to conceive in the future. Cancer treatments, including chemotherapy and radiation therapy, can impair ovarian function and lead to infertility, underscoring the need to explore FP options for these patients [1].

The necessity and urgency of FP in women with reproductive system malignancies cannot be overstated. Many cancer treatments are gonadotoxic, resulting in diminished ovarian reserve or complete loss of fertility. This reality necessitates close collaboration between oncologists and reproductive specialists to deliver comprehensive care that includes counselling and options for FP. Techniques such as oocyte cryopreservation, embryo freezing and ovarian tissue preservation are vital for women who wish to retain the possibility of biological motherhood following cancer treatment. Studies indicate that a significant proportion of women express a desire to have children post-treatment, yet many receive inadequate information about FP options before initiating cancer therapy [1,2]. This communication gap can cause emotional distress and regret, emphasizing the importance of integrating discussions about FP into cancer care protocols.

Assisted reproductive technology (ART) encompasses a range of methods designed to facilitate conception and support FP. These include procedures such as *in vitro* fertilization (IVF), intracytoplasmic sperm injection (ICSI) and embryo freezing, all of which play a pivotal role in enabling women with cancer to conceive after treatment. Advances in ART over the past few decades have significantly improved the success rates of these procedures, making them more accessible and effective for women facing fertility challenges due to malignancies. For example, the use of cryopreserved oocytes and embryos has become standard practice, allowing women to delay childbearing until completion of cancer treatment [3,4]. Furthermore, ongoing research into optimizing ART protocols and exploring innovative techniques – such as ovarian tissue transplantation and *in vitro* follicle maturation – continues to enhance prospects for FP in this population [2,5]. Thus, the role of ART in preserving fertility for women with reproductive system cancers is crucial, not only for supporting individual reproductive choices but also for improving overall quality of life and psychological well-being post-treatment.

## 1. Current status of FP in female patients with reproductive system malignant tumours

### 1.1. Mechanisms of fertility impairment

**Figure 1.**
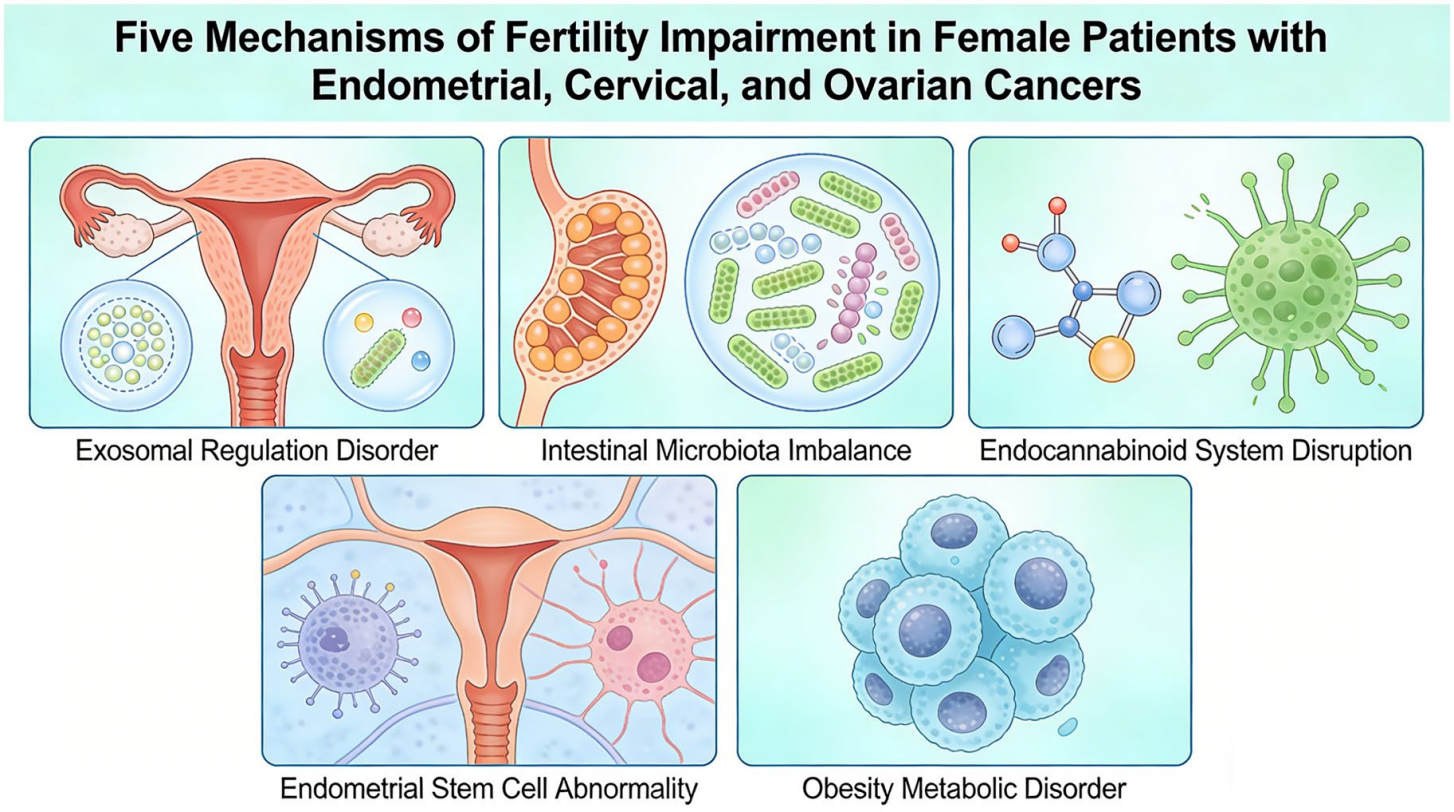
Schematic diagram of damage mechanisms.

Fertility impairment in female patients with malignant tumours, such as endometrial, cervical and ovarian cancers, involves multifaceted mechanisms linked to cellular dysregulation, hormonal imbalances and systemic disruptions, as shown in Figure 1. First, Exosomes, critical mediators of intercellular communication, carry genetic and proteomic cargo that regulate key reproductive processes including folliculogenesis, oogenesis and embryo implantation; their dysregulation in gynaecological cancers can alter signalling pathways, contributing to reproductive dysfunction [6]. Second, gut microbiota imbalances may act as an endocrine disruptor, indirectly impairing fertility through immune and metabolic perturbations, particularly in endometrial cancer [7]. Third, the endocannabinoid system, which plays a crucial role in folliculogenesis, fertilization and pregnancy progression, can be disrupted in cancers like ovarian and endometrial cancer, leading to hormonal imbalances and reproductive dysfunction [8]. Additionally, abnormalities in endometrial stem/progenitor cells, responsible for cyclic regeneration and differentiation of the endometrium, may impair tissue repair and embryo implantation in conditions such as endometrial cancer, contributing to infertility [9]. Finally, obesity, a risk factor for malignancies like endometrial cancer, exacerbates fertility impairment through metabolic dysfunctions such as insulin resistance and inflammation, which disrupt reproductive hormone balance and increase pregnancy complications [10]. Collectively, these interconnected mechanisms underscore the complexity of fertility impairment in female cancer patients, though further research is warranted to establish causality and refine therapeutic strategies.

**Figure 2.**
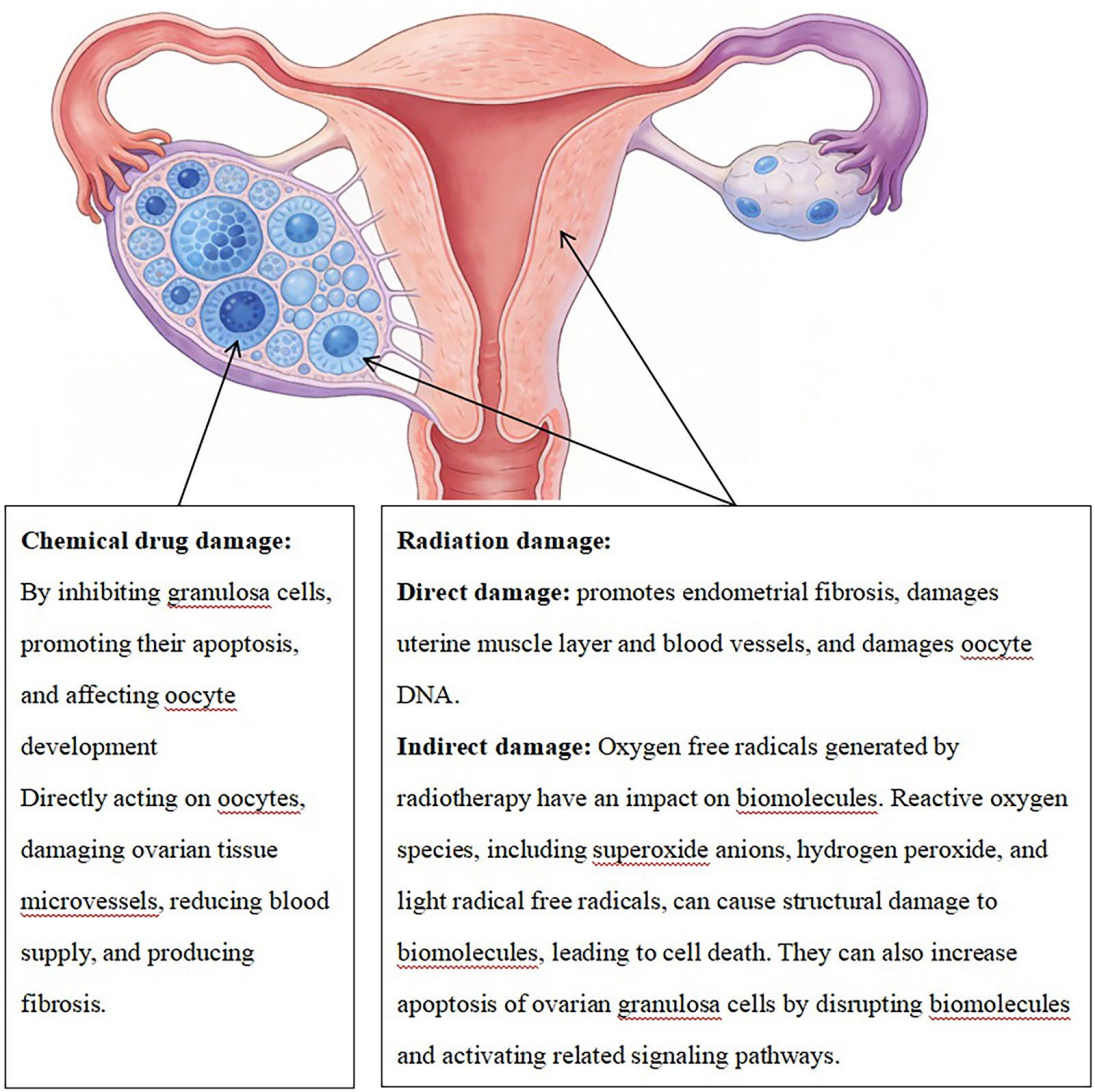
Schematic diagram of reproductive system damage caused by radiotherapy and chemotherapy.

On the other hand, the gonadal toxicity of tumour treatments, including chemotherapy, radiotherapy and surgical interventions, is also a significant cause of impaired fertility in female reproductive system cancers, as shown in Figure 2. First, chemotherapeutic agents, particularly alkylating agents, which can directly damage follicles, induce widespread follicular depletion and loss, leading to diminished ovarian reserve or premature ovarian insufficiency (POI). This is often reflected clinically by a significant decline in anti-Müllerian hormone (AMH) levels [11–13]. Second, radiotherapy can directly harm the ovarian parenchyma. The ovary is an extremely sensitive organ to radiotherapy. Even receiving a relatively low dose of radiotherapy may lead to impaired or complete loss of ovarian function, resulting in hormonal disorders and infertility. This is a significant cause of ovarian dysfunction and premature menopause in young patients [14–16]. Especially pelvic or whole-body irradiation can cause irreversible damage to ovarian function and uterine integrity, affecting follicular development and endometrial receptivity. The side effects of pelvic radiotherapy are not limited to the ovaries, but also affect the uterus and endometrium, thereby influencing future pregnancy outcomes [17,18].

Third, Surgical treatments such as oophorectomy may cure diseases, but organ resection can directly reduce the follicular pool and surgical procedures may also damage the function of reproductive organs, such as impairing the blood supply to the ovaries, thereby threatening fertility. Although surgical techniques aimed at sparing reproductive organs and preserving fertility have been developed, their indications are usually limited to early-stage well differentiated low-grade tumours or women with low malignant potential [19–21]. The surgical approach itself may also influence ovarian preservation outcomes [22].

Furthermore, the disease process can also be a direct contributor, as the presence, progression, or recurrence of certain ovarian tumours (e.g. non-dysgerminomas) can compromise ovarian tissue and function [23]. Furthermore, allogeneic haematopoietic stem cell transplantation (HSCT) introduces an additional risk factor, as graft-versus-host disease (GVHD) is associated with decreased fertility [13]. Even FP procedures carry inherent risks; ovarian tissue cryopreservation and transplantation are associated with follicular loss during the freezing-thawing process and there exists a concern regarding the potential reintroduction of malignant cells *via* ovarian tissue transplantation in diseases like haematological malignancies [13]. Finally, the gonadotoxic effects of cancer treatment compound the natural, age-related decline in ovarian reserve, collectively resulting in a significantly shortened reproductive window and an elevated risk of infertility for survivors [24,25]. These findings underscore the urgent need for comprehensive oncological fertility counselling and the development of advanced FP strategies before initiating cancer treatment.

### 1.2. Clinical demand and challenges of FP

The clinical demand for FP in female patients with malignant tumours is substantial and growing, driven by improved long-term survival rates and the well-documented gonadotoxic effects of many anticancer therapies [26–28]. This concern affects a significant proportion of young patients; for instance, in Hungary, an average of 2,066 women under 40 are diagnosed with cancer annually, with approximately two-thirds facing potential fertility impairment due to treatment [26]. Key populations include young women with breast cancer, lymphoma, leukaemia and gynaecological malignancies such as cervical and endometrial cancers [29,30]. Established and experimental FP options aim to address this demand. Embryo cryopreservation is a well-established method but requires a delay in cancer treatment of 2–4 weeks for ovarian stimulation and the availability of a partner or donor sperm [31,32]. Oocyte vitrification has become a robust option, especially for women without a partner, while OTC is often the only feasible option for prepubertal girls and in urgent clinical situations, despite concerns about the potential risk of reimplanting malignant cells [33]. The use of gonadotropin-releasing hormone agonists for ovarian protection during chemotherapy remains a subject of ongoing debate regarding its efficacy [34].

Despite clinical guidelines from societies like ESHRE and ASCO recommending timely FP counselling for all eligible patients, significant disparities in access and utilization persist globally [28,35–37]. Real-world uptake remains low; a U.S. study found that only about 5.5% of eligible women received an FP evaluation in 2016 [38], and a Polish study reported that only 19% of patients were encouraged to see a fertility specialist [39].

In sub-Saharan Africa and Latin America, only 15% of reproductive-age cancer patients have access to FP services, compared to 78% in high-income countries [40]. Access is influenced by factors such as patient age, cancer type, geographical region and significant barriers including lack of information among both patients and professionals, limited cryopreservation facilities, high costs, and cultural stigma around cancer and infertility, and the urgency to begin cancer treatment [38,41–43]. The psychological impact of these unmet needs is considerable. Failure to receive FP counselling is associated with higher levels of decision regret among survivors, and patients’ choices are often affected by practical constraints like lack of time and emotional distress [44].

Special considerations are paramount for specific subgroups. For paediatric and adolescent patients, ethical considerations are heightened, and OTC is typically the primary option [45,46]. For women with cervical cancer, management strategies range from fertility-sparing surgeries (e.g. radical trachelectomy) in early-stage disease to FP using oocyte/embryo freezing for future surrogacy in advanced cases requiring pelvic radiotherapy [30,47]. Future directions to better meet this clinical demand include optimizing techniques like OTC and *in vitro* follicle maturation, developing an artificial ovary to eliminate reseeding risk, and establishing streamlined, multidisciplinary oncofertility care pathways to improve coordination between oncology and reproductive medicine teams [48,49].

FP for patients with gynaecologic malignancies requires a well-coordinated, multidisciplinary approach to address the complex medical, surgical and psychosocial needs of these patients [50,51]. The primary goal is to integrate FP seamlessly into the oncologic care pathway without compromising cancer treatment outcomes.

A significant challenge is the need for timely and effective coordination between the patient, gynaecologic oncologists, reproductive endocrinologists and often paediatric specialists for younger patients [52]. Delays in referral or a lack of established protocols can limit patient access to FP options. This coordination is critical for urgent cases where cancer treatment must begin swiftly, necessitating rapid decision-making between options like emergency IVF and OTC [53].

The choice of FP strategy itself depends on coordinated risk assessment. For instance, while OTC followed by transplantation is a valuable option, its safety must be evaluated on a case-by-case basis for patients with malignancies like leukaemia or ovarian cancer due to the risk of reintroducing malignant cells [54]. This requires close collaboration between oncologists and fertility specialists to determine the safest approach.

The surgical and anaesthetic management of fertility-sparing procedures, such as radical trachelectomy or the emerging field of uterine transplantation, also demands a highly coordinated peri-operative team to manage the specific physiological demands and optimize outcomes [55,56].

Beyond the clinical coordination, effective care must address the patient’s long-term survivorship journey. Young women who undergo FP, particularly those with stored ovarian tissue, face complex emotional and ethical dilemmas regarding future family building [57]. A coordinated survivorship plan that includes access to psychosocial support and clear information about the use of stored tissue is essential.

To overcome these challenges, the development of integrated oncofertility programs is key. These programs, as envisioned by multidisciplinary consortia, aim to build a community of care by sharing knowledge rapidly and establishing best practices across medical specialties, scientists and scholars in related fields. Future efforts should focus on standardizing referral pathways, improving interdisciplinary communication and providing comprehensive support that addresses both the immediate FP decisions and the long-term reproductive and emotional needs of cancer survivors.

## 2. Mature ART for FP

### 2.1. Oocyte cryopreservation

With improving cancer survival rates, FP has become an integral component of comprehensive oncologic care. Oocyte cryopreservation has emerged as a pivotal strategy for FP, offering a viable option particularly for women with medical or social reasons [58]. Recent studies emphasize individualized stimulation protocols, including random-start or dual-stimulation cycles, to accommodate urgent cancer therapy timelines [59].oocyte cryopreservation is a critical consideration for young women diagnosed with various reproductive cancers before initiating gonadotoxic therapies. This includes patients with early-stage cervical cancer (e.g. stage Ib1-IIb), endometrial cancer, and ovarian pathologies such as borderline ovarian tumours (BOTs) [60]. Furthermore, women with hereditary breast and ovarian cancer (HBOC) syndrome, particularly BRCA1 mutation carriers, represent a unique high-risk group. Evidence suggests BRCA1 mutations are associated with occult primary ovarian insufficiency and a poorer ovarian response to stimulation, underscoring the importance of early FP consultation and intervention in this population [61].

The success of oocyte cryopreservation hinges on effective controlled ovarian stimulation (COS) to retrieve a sufficient number of mature oocytes. For patients with hormone-sensitive cancers, such as breast cancer, COS protocols co-administered with letrozole or tamoxifen are employed to mitigate the risk of elevated os exposure [62–64]. Reported outcomes are promising; one study of patients with gynaecologic malignancies undergoing COS for FP reported a median yield of 11 oocytes, with subsequent utilization of cryopreserved oocytes/embryos resulting in live births in 58.8% of returning patients [60]. Current evidence indicates that the likelihood of achieving a live birth using frozen oocytes is strongly associated with the woman’s age at oocyte retrieval, with optimal results observed in women under 35 years [65]. Studies have shown that freezing a sufficient number of oocytes – ideally between 14 and 20 – improves the chances of a successful pregnancy, as the number of retrieved oocytes directly correlates with live birth probability [66]. For example, a retrospective cohort study reported that women who underwent elective oocyte cryopreservation had a cumulative live birth rate of approximately 32.4% after thawing and using their oocytes, highlighting the importance of age and the number of frozen oocytes [67]. Furthermore, research suggests that while live birth rates from frozen oocytes may be lower than those from fresh oocytes, outcomes are comparable when adjusted for age and other factors [68]. However, it is critical to note that oocyte cryopreservation does not guarantee future pregnancy, and patients should receive counselling on realistic expectations regarding success rates and potential risks [69].

Optimizing the handling of immature oocytes retrieved during COS is another key area. Research indicates that performing *in vitro* maturation of germinal vesicle and metaphase I oocytes prior to cryopreservation (‘fresh IVM’) yields significantly higher combined survival and maturation rates (63.8%) compared to cryopreserving immature oocytes and maturing them post-thaw (‘post-thaw IVM’, 33.3%). This strategy is therefore recommended to maximize the reproductive potential of all retrieved gametes [70,71].

Integrating FP into oncologic surgery requires careful planning. For patients with ovarian malignancies where transvaginal puncture risks capsular rupture and tumour dissemination, alternative methods have been developed. *Ex vivo* or extracorporeal oocyte retrieval, performed on the oophorectomized specimen immediately after surgical removal, allows for oocyte harvest without compromising oncologic safety [72]. Similarly, *in vivo* retrieval during surgical staging for gynaecologic cancers has been shown to be feasible without causing significant treatment delays [73].

Safety monitoring remains paramount. In patients with BOTs undergoing oocyte retrieval after fertility-sparing surgery, cytological examination of atypical cystic puncture fluid can lead to the diagnosis of recurrence, highlighting the need for vigilance during the procedure [74].

Despite its established role, oocyte cryopreservation faces several limitations. It is generally recommended for women under 40 years of age [75]. While oocyte cryopreservation is no longer considered experimental, other adjunctive methods like ovarian tissue cryopreservation or gonadotropin-releasing hormone agonist co-treatment during chemotherapy are still under investigation [76]. A significant barrier identified in practice is the gap in knowledge and coordination between oncology and reproductive medicine teams. Surveys indicate that many non-specialist providers lack detailed FP knowledge, and fertility specialists are often not integrated into multidisciplinary tumour boards, potentially leading to under-referral of eligible patients [75,77].

### 2.2. *In vitro* maturation (IVM) of oocytes

For patients with gynaecological malignancies, such as ovarian cancer, who require oophorectomy, IVM of oocytes has emerged as a significant and promising option due to its ability to avoid ovarian stimulation, shorten the treatment cycle and maintain a favourable safety profile [78,79]. Its core application involves retrieving immature oocytes from unstimulated or surgically resected ovaries, maturing them under laboratory conditions, followed by fertilization and cryopreservation of the resulting embryos, thereby preserving fertility potential for patients after completing anticancer therapy [80]. Successful clinical practice has been documented: a live birth was achieved from cryopreserved embryos derived from oocytes that underwent IVM following oophorectomy in a patient with stage IIIC ovarian cancer, representing the first reported pregnancy and live birth from this approach [81]. Furthermore, IVM has been considered for FP in patients with leukaemia and prior to procedures such as ovarian transposition or endometrioma excision necessitated by cancer [82].

IVM offers multiple advantages for oncology patients. First, it avoids ovarian stimulation entirely, eliminating the need for exogenous gonadotropins and thereby fundamentally circumventing the risk of ovarian hyperstimulation syndrome (OHSS), which is particularly crucial for cancer patients [78]. Second, the treatment cycle is short and immediately feasible: it requires no prolonged hormonal preparation, making it a key advantage for patients needing to commence anticancer treatments (e.g. chemotherapy, radiotherapy) promptly [83]. Third, it is applicable to patients with diminished ovarian reserve or poor responders to ovarian stimulation.

However, its clinical application nonetheless faces several challenges. Primarily, the developmental potential and embryo quality of IVM oocytes are generally inferior to those of *in vivo*-matured oocytes, which may be associated with inadequate cytoplasmic maturation, leading to lower implantation and pregnancy rates compared to conventional ovarian stimulation regimens in normo-ovulatory women [80,81]. Secondly, oocyte maturation *in vitro* is a complex biological process susceptible to meiotic errors, warranting considerable caution in its clinical application [82]. Although techniques are continually optimized (e.g. the biphasic CAPA-IVM system can improve maturation rate), IVM has not yet become a mainstream conventional treatment and is more often considered an alternative option for specific circumstances [83]. Further improvement in its success rates depends on ongoing research into culture systems, supplements and patient selection criteria [84]. Therefore, despite existing successful cases, more evidence and continuous technological refinement are required before establishing IVM as a routine FP strategy for oncology patients [82]. Future investigations into different culture media, additives (such as growth hormone) and follicular priming regimens hold promise for further enhancing the success of IVM in this patient population [84,85].

### 2.3. Embryo cryopreservation

Embryo cryopreservation is a well-established and standard method for FP in female patients diagnosed with cancers of the reproductive system [86,87]. This technique is widely available and primarily indicated for women who have a male partner or are willing to use donor sperm, and for whom a delay in initiating adjuvant cancer therapy is acceptable [88]. It is applicable for patients with various gynaecologic malignancies. For instance, conservative surgical approaches combined with FP strategies can be considered for early-stage cervical cancer, endometrial cancer and ovarian tumours [88]. Furthermore, embryo cryopreservation is a key option for women with hereditary breast and ovarian cancer syndrome [61]. Decisions are individualized based on the patient’s age, ovarian reserve, cancer type and stage and treatment timeline [89].

The process requires controlled ovarian stimulation to retrieve oocytes. For patients with hormone-sensitive tumours like breast cancer, specific COS protocols are employed to minimize risks; co-administration of tamoxifen or aromatase inhibitors with gonadotropins has been reported to be successful [90,91]. Following fertilization, the resulting embryos are cryopreserved. Embryo cryopreservation is recognized as the only established method of female FP for young cancer patients. Data indicates successful outcomes, with one study reporting live births in 58.8% of patients with gynaecologic malignancies who returned to use their cryopreserved oocytes or embryos [60], another study indicated notably high clinical pregnancy rates for embryo cryopreservation, with approximately 49% reported for patients using cryopreserved embryos [92]. Moreover, cryopreservation allows for delayed embryo transfer until the patient’s health or circumstances are more favourable, optimizing the chances of successful implantation and live birth [93]. Research on the safety of cryopreserved embryos has found no increased risk of adverse perinatal outcomes compared to fresh transfers [94]. However, it is important to note that while overall success rates are promising, individual outcomes vary based on factors such as maternal age, ovarian reserve and embryo quality at freezing [95].

However, women with BRCA mutations present a unique FP challenge due to their cancer risk and potential for diminished ovarian reserve. Research suggests BRCA1 mutations are associated with occult primary ovarian insufficiency, which may manifest as a poorer response to ovarian stimulation [96]. However, a comparative study found that when adjusted for factors like age and anti-Müllerian hormone level, the outcomes of embryo cryopreservation cycles in BRCA carriers were comparable to those of non-carriers, supporting its feasibility in this population [61].

Despite its established role, embryo cryopreservation has limitations. It is not an option for prepubertal patients, women without a partner unwilling to use donor sperm, or when cancer treatment is too urgent to permit delay. Success is dependent on the patient’s age and ovarian reserve at the time of stimulation [89]. A significant barrier in clinical practice is the gap in knowledge and coordination between oncology and reproductive medicine teams. Surveys indicate that many non-specialist providers lack detailed FP knowledge, and fertility specialists are often not integrated into multidisciplinary tumour boards, which can hinder patient referral and access to this standard option [75].

In summary, embryo cryopreservation remains a fundamental and effective standard of care for FP in eligible women with reproductive system cancers. Ongoing efforts to optimize patient selection, integrate FP discussions into multidisciplinary oncology care, and improve provider education are essential to ensure equitable access and optimal outcomes.

### 2.4. Ovarian tissue cryopreservation (OTC) and transplantation

OTC has emerged as a significant FP strategy for women of reproductive age facing gonadotoxic treatments, particularly those diagnosed with gynaecologic malignancies. This technique involves the surgical retrieval and cryopreservation of ovarian cortical tissue, which contains primordial follicles, for subsequent transplantation after cancer remission. Its application in patients with gynaecologic tumours requires careful consideration of indications, techniques, outcomes and evolving strategies [97].

OTC is primarily indicated for young women and adolescents at risk of premature ovarian insufficiency due to planned cancer therapies, who cannot delay treatment to undergo ovarian stimulation for oocyte cryopreservation [89,98]. For patients with borderline ovarian tumours, OTC is considered a viable FP option alongside oocyte cryopreservation and fertility-sparing surgery [99]. However, its application in invasive ovarian cancer remains highly controversial and is generally not recommended outside experimental protocols due to the potential risk of reintroducing malignant cells upon future tissue transplantation [100,101]. The procedure is contraindicated for cancers with a high risk of ovarian metastasis, such as leukaemia, neuroblastoma and Burkitt’s lymphoma [98,102]. A multidisciplinary team discussion is essential for patient selection, which typically restricts OTC to women under 35 years of age with a good prognosis [103].

The standard procedure involves laparoscopic retrieval of ovarian cortical strips, which are then cryopreserved using either slow-freezing or vitrification protocols. A minimally invasive alternative, vaginal natural orifice transluminal endoscopic surgery (vNOTES), has been introduced for ovarian biopsy, offering potential benefits like reduced postoperative pain and faster recovery, enabling quicker initiation of cancer therapy [98]. The principal safety concern is the possible presence of malignant cells in the cryopreserved tissue. This risk precludes the use of OTC for patients with ovarian cancer and certain other malignancies, as transplantation could lead to disease recurrence [100,101]. To mitigate this risk, innovative strategies such as using decellularized ovarian scaffolds are under investigation to create an ‘artificial ovary’ free of malignant cells [104].

OTC followed by autotransplantation has proven successful in restoring fertility. A live birth has been reported following heterotopic transplantation of cryopreserved tissue in a patient with early-stage ovarian cancer [101]. In a cohort of patients with malignant and borderline ovarian tumours, OTC was one of the FP methods employed, demonstrating its integration into clinical practice for selected cases [103]. A recent retrospective study [105] reported that 12/38 (31.6%) reproductive-age endometrial cancer patients (stage IA, grade 2) achieved live birth after ovarian tissue transplantation, with no recurrence during 5-year follow-up, supporting the feasibility of fertility-sparing approaches in selected patients. Overall, ovarian stimulation for oocyte/embryo cryopreservation appears safe in gynaecologic cancer patients, with no significant difference in long-term oncologic outcomes compared to those who did not undergo FP procedures [106]. The success of transplantation is influenced by the density of primordial follicles in the grafted tissue and the patient’s age at cryopreservation [101]. However, some studies have pointed out that the risk of reintroducing malignant cells is 2-5% for hormone-sensitive tumours (e.g. endometrial cancer) vs. <1% for non-hormone-sensitive tumours (e.g. cervical squamous cell carcinoma) [107,108]. Previous reviews failed to account for tumour stage – stage IA tumours have negligible risk, while stage II-IV tumours have a 7.8% recurrence risk post-transplantation [101,109].

The major limitation of OTC is the mandatory invasive surgery for both tissue harvesting and later transplantation, coupled with the risk of malignant cell reintroduction. Future directions aim to overcome these hurdles. *In vitro* folliculogenesis is a promising area of research that seeks to mature primordial follicles from cryopreserved tissue entirely in the laboratory, eliminating the need for reimplantation and its associated risks [102]. As noted, tissue engineering approaches, such as recellularizing decellularized ovarian scaffolds with isolated follicles, are being explored to create a biocompatible, cancer-free transplant [104]. Furthermore, standardizing care pathways and improving knowledge among healthcare providers are necessary to enhance patient access and outcomes [75]. Recent advancements focus on improving tissue viability through optimized cryoprotectants, whole-ovary cryopreservation research, and robotic-assisted harvesting to minimize ischaemia time [110,111]. Continued research and multidisciplinary collaboration are essential to refine patient selection, improve techniques and develop safer alternatives for fertility restoration in cancer survivors [89].

In summary, OTC represents a promising component of FP for selected reproductive-aged women with gynaecologic tumours, particularly BOTs and non-ovarian malignancies. Continued innovation in surgical techniques and tissue engineering, coupled with rigourous patient selection and multidisciplinary collaboration, is essential to optimize outcomes and minimize risks.

### 2.5. Gonadotropin-releasing hormone agonists (GnRHa)

GnRHa serve as a component of fertility-sparing treatment, primarily for early-stage endometrioid endometrial cancer and atypical endometrial hyperplasia, and are frequently employed within combination regimens rather than as monotherapy. A qualitative systematic review [112] demonstrates that fertility-sparing treatment, primarily using progestin-based regimens, is a feasible option for selected young patients with FIGO stage IA, grade 2 endometrioid endometrial cancer. The evidence indicates a favourable initial complete response rate; however, it is accompanied by a considerable risk of disease recurrence. While successful pregnancies are achievable, outcomes vary. The findings highlight the necessity of rigourous patient selection, close surveillance involving regular endometrial sampling, and a multidisciplinary approach to balance oncologic safety with reproductive goals. A significant application is as a salvage strategy for patients exhibiting progestin resistance, where GnRHa-based re-treatment (alone or combined with a levonorgestrel-releasing intrauterine system or an aromatase inhibitor) can achieve a complete response (CR) rate of approximately 90.2% [113]. For obese patients (BMI ≥30 kg/m^2^), GnRHa-based regimens represent a viable option due to a lesser impact on weight gain compared to progestin therapy, with weight loss of >10% being associated with improved CR, lower recurrence and better pregnancy rates [112]. However, oncological outcomes require careful consideration; despite high initial CR rates, recurrence remains a concern, with one study reporting a 34.5% recurrence rate after a median follow-up of 36 months following CR [113] and another suggesting a potentially higher recurrence risk with the GnRHa plus levonorgestrel-releasing intrauterine device (LNG-IUD) combination compared to other regimens [114]. Reproductive outcomes are variable: while successful pregnancies and live births have been documented, a systematic review indicated that regimens combining GnRHa with LNG-IUD or letrozole may be associated with lower live birth rates [115]. Contemporary management is increasingly individualized, incorporating prognostic factors such as weight management and molecular classification; for instance, integrating immune checkpoint inhibitors with hormonal therapy may be an effective fertility-preserving approach for patients with mismatch repair deficiency [116].

In summary, the use of GnRHa during chemotherapy remains a complementary and controversial FP strategy. Recent meta-analyses suggest it may reduce the risk of premature ovarian insufficiency and improve post-treatment pregnancy rates, particularly in breast cancer patients. However, it is not recommended as a standalone method but rather as an adjunct to cryopreservation techniques. Current guidelines support its use within specific clinical contexts while acknowledging the need for further randomized evidence.

**Figure 3.**
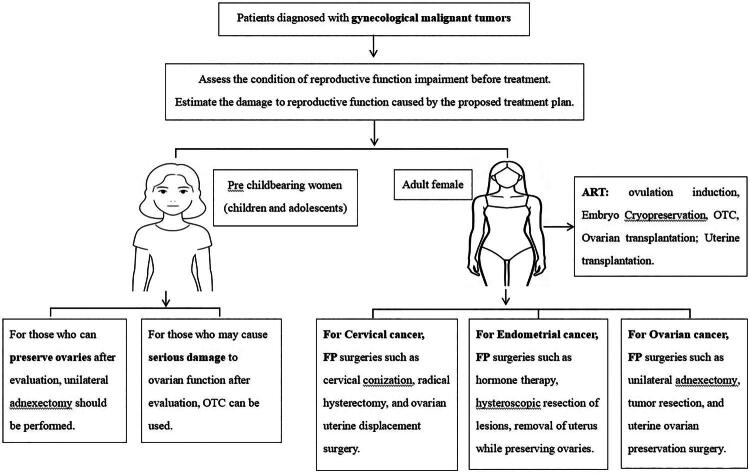
Schematic diagram of FP decision-making process for gynaecological malignant tumour patients. FP: fertility preservation, OTC: ovarian tissue cryopreservation, ART: assisted reproductive technology.

The decision-making process for FP in patients with gynaecological malignant tumours is shown in Figure 3.

## 3. Future directions and experimental approaches

The field of FP for gynaecological cancer patients is evolving from conventional assisted reproductive technologies towards integrated approaches in regenerative medicine and precision oncology. Key advances are focused on three interconnected domains: technological innovation, personalized treatment strategies and multidisciplinary collaboration.

Technological Innovations include refinements in OTC, such as enhanced vitrification protocols and the use of biomaterials to support revascularization post-transplant [117]. Experimental systems like *in vitro* maturation (IVM) of oocytes, microfluidic culture devices and ovarian organoid models are being developed to improve follicular survival and simulate the ovarian niche [118]. Research on ovarian stem cells and bioengineered artificial ovaries represents a promising frontier for patients with diminished ovarian reserve [119].

Personalized FP Strategies are increasingly guided by tumour molecular profiling (e.g. BRCA status, hormone receptor expression) to tailor ovarian stimulation protocols and assess oncological safety [120]. Parallel efforts aim to mitigate gonadotoxicity from novel anticancer agents (e.g. PARP inhibitors, immunotherapy) through adjuvants like GnRH agonists and *in vitro* toxicity screening using follicular or organoid cultures [121].

Multidisciplinary Integration is essential for optimizing FP pathways. Established oncofertility networks now incorporate tumour boards, reproductive specialists, genetic counsellors and psycho­logical support [122]. Long-term follow-up studies and AI-based predictive models are being em­­­ployed to evaluate reproductive outcomes, offspring health and potential epigenetic impacts of FP techniques [123].

Despite progress, challenges remain, including limited graft longevity after ovarian transplantation and the need for more robust *in vitro* follicle culture systems. Future directions may involve gene-editing tools to correct hereditary cancer mutations and ‘ovarian-on-a-chip’ platforms for real-time monitoring. Overall, the integration of experimental methods with clinical translation aims to achieve the dual goals of cancer survival and fertility restoration.

## 4. Ethical, legal and medicolegal implications

The field of FP in patients with gynaecological malignancies has witnessed significant advancements, accompanied by evolving ethical, legal and medicolegal considerations. Progress is primarily reflected in evidence-based decision-making, exploration of clinical consensus, and a heightened focus on patients’ multidimensional needs, as supported by recent literature.

### 4.1. Ethical controversies in oncofertility

From an ethical perspective, a key advancement is the quantification of treatment success rates, which is fundamental for shared decision-making and valid informed consent. For women with early-stage endometrial cancer desiring FP, conservative management (CMEC) is a crucial option. A 2022 systematic review and meta-analysis provided pivotal data, estimating that women undergoing progestin-based CMEC have a 26.7% chance of achieving pregnancy and a 20.5% chance of a live birth. This quantitative evidence is essential for ethically balancing oncological safety with reproductive aspirations. Furthermore, the same study found that a longer follow-up duration was positively associated with higher chances of pregnancy and live birth, underscoring the ethical obligation for long-term, meticulous surveillance [1]. Additionally, identifying risk factors for recurrence is an integral part of ethical care. Research indicates that a higher metabolic risk score (MRS) and insulin resistance (IR) are independent risk factors for recurrence in patients with atypical endometrial hyperplasia (AEH) and early endometrial cancer undergoing fertility-sparing treatment [3]. This highlights the ethical imperative for more cautious evaluation and stringent follow-up for high-risk patients.

Nevertheless, the intersection of oncology and ART raises profound ethical questions that extend beyond immediate clinical care.

First, posthumous reproduction. The fate of cryopreserved gametes, embryos, or ovarian tissue in the event of a patient’s death presents a significant ethical challenge. This issue touches upon the autonomy and prior expressed wishes of the deceased, the legal status of genetic material, the welfare of potential offspring, and the rights of surviving partners [124]. Current ethical frameworks emphasize the paramount importance of obtaining detailed, informed consent and legally binding advance directives prior to FP procedures, explicitly outlining the patient’s wishes for the disposition of stored materials in case of death. Posthumous reproduction is legal in 7 countries (e.g. France, Canada) but prohibited in 23 (e.g. Germany, China). Key ethical debates focus on the rights of the deceased (autonomy vs. family wishes) and the psychological impact on children – 80% of children born *via* posthumous reproduction report identity confusion by age 12 [125,126]. Second, regarding gestational surrogacy. For cancer survivors who have undergone radical hysterectomy, resulting in absolute uterine factor infertility, surrogacy may represent the only pathway to genetic parenthood [127]. This practice is at the centre of global ethical and legal debates concerning commercialism, potential exploitation, the rights of gestational carriers and legal parentage determination [128]. Regulatory landscapes vary dramatically across jurisdictions, from complete prohibition to regulated commercial frameworks, adding complexity to cross-border arrangements [129]. Third, informed consent in the era of precision medicine. The integration of molecular classification into gynaecologic oncology, particularly for endometrial cancer, is fundamentally altering the informed consent process [130,131]. For instance, counselling a patient with early-stage endometrial cancer about fertility-sparing hormonal therapy now may require discussion of tumour molecular subtypes (e.g. POLE-mutant, p53-abnormal), which carry distinct prognostic implications for recurrence risk [132]. This necessitates that patients comprehend complex biologic data to make truly informed decisions about conservative management, thereby increasing the demands on the consent process.

### 4.2. Legal, professional guideline frameworks and medicolegal implications

A multi-layered framework of professional guidelines and evolving legal standards guides FP practice to protect patient welfare and define the standard of care. Recommendations from bodies such as the American Society of Clinical Oncology (ASCO) and the American Society for Reproductive Medicine (ASRM) form a cornerstone of global practice. ASRM 2022 guidelines mandate that oncologists discuss FP options within 48 h of cancer diagnosis [133], while ASCO 2023 guidelines recommend insurance coverage for medically indicated FP [134]. These complement the UK Montgomery ruling (2015) by emphasizing proactive counselling rather than passive consent. A comprehensive review [130] elucidates the revolutionary potential and broad spectrum of applications of CRISPR-Cas systems, ranging from human genome editing and therapeutic interventions to agricultural and environmental uses. It critically details the persistent technical limitations, including off-target effects and delivery challenges, and discusses potential hazards such as unintended ecological consequences. The authors conclude that responsible translation of this powerful technology into practice is of paramount importance, necessitating a robust, forward-looking bioethical framework and stringent regulatory oversight to mitigate risks and navigate the complex societal implications. A critical appraisal confirms that cfDNA-based NIPT is a highly accurate screening test for common foetal aneuploidies, though a positive result requires confirmatory invasive testing. Its application, especially for monogenic disorders, must be guided by comprehensive pre-test counselling and a clear ethical-legal framework to manage its limitations and implications [135].

Core principles enshrined in these guidelines include: (1) the necessity of multidisciplinary team (MDT) collaboration, ensuring early involvement of both reproductive specialists and gynaecologic oncologists [135]; (2) timely referral to initiate FP counselling without delaying life-saving cancer therapy [136] and (3) the prioritization of proven FP techniques [106]. Surveys, such as one conducted nationally in Spain, highlight that adherence to and standardization of these guidelines are essential to ensure equitable and high-quality care across different treatment centres [136]. Disease-Specific Consensus Criteria: For each gynaecologic malignancy, ethical and legal ‘safety boundaries’ for FP are continuously refined through clinical research and codified in practice guidelines. In cervical cancer, specific radiologic and pathologic criteria (e.g. tumour size <2 cm, absence of lymphovascular space invasion) serve as critical thresholds for determining eligibility for procedures like radical trachelectomy [137,138]. For endometrial cancer, conservative management with progestin therapy is recommended only for a highly selected cohort with low-grade, early-stage disease confined to the endometrium, mandating rigourous surveillance [139]. These criteria provide a medico-legal safeguard for clinicians.

Regarding legal and medicolegal implications, progress centres on adherence to guidelines, consensus on eligibility criteria, and risk assessment of new technologies. A 2023 survey among Swedish gynaecologists and gynaecological oncologists revealed variability in clinical practice and potential legal pitfalls. While a majority supported CMEC, significant disagreement existed regarding specific eligibility criteria; for instance, over one-third of respondents disagreed with offering CMEC to patients with known fertility problems, recurrent miscarriages, or previous children [2]. Such inconsistency in clinical judgesment can lead to disparate standards of care across institutions, potentially giving rise to legal disputes. The survey also suggested that oncologists were more involved in patient management than fertility specialists, indicating a need to optimize multidisciplinary collaboration to ensure comprehensive and legally sound decision-making [2]. Concerning technological advances, robotic radical trachelectomy has emerged as a minimally invasive option for FP in early-stage cervical cancer [14]. While initial data suggest its safety, the widespread adoption of this technique necessitates established protocols, thorough patient counselling regarding potential obstetric outcomes (e.g. preterm labour risk), and ongoing evaluation – all critical components of medicolegal risk management [14].

### 4.3. Psychosocial well-being and reproductive health rights

The comprehensive attention to patients’ psychosocial well-being and reproductive health rights represents a significant ethical advancement. Studies show that young women with cervical cancer experience substantial reproductive concerns and fertility anxiety, which can be categorized into distinct profiles, even when fertility-sparing treatments are available [5]. This underscores that ethical care must extend beyond technical success to integrate psychosocial support and respect for reproductive autonomy. Concurrently, advancements in basic science, such as elucidating the role of ferroptosis in female reproductive disorders, offer potential future therapeutic targets for FP strategies [140]. This prompts forward-looking ethical considerations regarding balancing risks and benefits in clinical research of novel therapies and ensuring equitable access to future resources.

### 4.4. Medicolegal and medical malpractice risks

The FP pathway is fraught with potential pitfalls that can lead to medical disputes. Key areas of medicolegal risk include: (1) Inappropriate Patient Selection: This constitutes a primary source of malpractice liability. Offering fertility-sparing treatment to patients who do not meet strict oncologic criteria (e.g. larger tumour volume, high-risk histology) may be deemed negligent if it results in disease recurrence or progression [136]. Adherence to established guidelines and meticulous preoperative imaging assessment are essential risk mitigation strategies [138]. (2) Inadequate Informed Consent: Failure to comprehensively disclose all treatment alternatives (including radical and fertility-sparing options), their associated oncologic risks, success rates of FP, potential obstetric complications (e.g. preterm birth after trachelectomy), and the future likely need for ART can render the consent process legally deficient [88]. Consent must also cover the specific techniques planned, such as controlled ovarian stimulation for oocyte cryopreservation [106]. A study [141] showed that only 22.7% of cancer patients of childbearing age have received information about the risk of infertility caused by treatment, and among patients with reproductive system cancer, this proportion was only 59.3%. (3) Delay in Cancer Treatment: A fundamental ethical and legal principle is that FP interventions must not unduly postpone definitive cancer therapy. Although studies suggest that time to subsequent cancer treatment may be marginally longer for patients undergoing ovarian stimulation, no significant negative impact on oncologic outcomes has been consistently demonstrated [106,142]. However, an unreasonable delay caused by poor coordination or procedural inefficiency could constitute serious medical negligence [143]. (4) Failure in Multidisciplinary Communication: Effective MDT collaboration is not only a best-practice standard but also a key risk management strategy. Breakdowns in communication between gynaecologic oncologists and reproductive endocrinologists can lead to suboptimal timing, inconsistent patient advice, or incomplete information sharing, ultimately harming patient care [136,144]. Clearly defined referral pathways and formal documentation of team discussions are vital for medicolegal protection. (5) The risk associated with inadequate follow-up and management: Following successful fertility-sparing treatment, long-term and rigourous follow-up is imperative. This includes regular endometrial surveillance to detect recurrence early, and the timely recommendation for definitive surgery, such as hysterectomy, once childbearing is complete [145]. A retrospective study [146] highlights significant gaps in oncofertility care for breast cancer patients, revealing that while a majority of young patients were referred for reproductive counselling, a considerably lower proportion proceeded with fertility preservation methods such as oocyte or embryo cryopreservation. The findings emphasize that patient decisions were influenced by factors including disease stage, treatment urgency and personal priorities. The authors conclude that systematic integration of timely and personalized fertility counselling into standard oncology care is crucial to empower patients and improve reproductive outcomes. Interruption of the follow-up protocol or negligence in recognizing signs of recurrence can lead to delays in necessary treatment, potentially resulting in serious consequences and giving rise to medicolegal disputes.

In summary, the ethical, legal and medicolegal progress in FP for gynaecological malignancies is evidenced by enhanced evidence-based support, ranging from macro-level success rates to micro-level risk factors [145,147], ongoing efforts to standardize clinical practice and multidisciplinary collaboration [146], and a concurrent focus on patient psychosocial needs and the risks associated with emerging technologies [148,149]. These developments collectively guide clinical practice towards more responsible, personalized and rights-protective approaches. Future development requires sustained dialogue among ethicists, legal experts, clinicians and policymakers to construct a robust support system that honours patient autonomy while effectively managing associated risks.

## 5. Clinical, public health and policy value

For female patients with malignant tumours of the reproductive system and fertility needs, assisted reproductive technology (ART) plays a key role in FP strategies, and its value is reflected in multiple levels of clinical, public health and policy.

### 5.1. Clinical value: optimizing reproductive outcomes and ensuring safety

The primary clinical value of ART lies in its capacity to facilitate pregnancy and live births for cancer survivors who have undergone fertility-preserving therapy. For women with early-stage endometrial cancer (EC) or atypical endometrial hyperplasia (AEH) treated conservatively with progestins, achieving a pregnancy can be challenging. Evidence indicates that ART significantly improves reproductive outcomes in this population. One review reported that the pregnancy rate was markedly higher in an ART group compared to a natural conception group (80.0% vs. 43.2%) [150]. Another study confirmed that utilization of ART was positively associated with a higher pregnancy rate following conservative management [151]. Specifically, *in vitro* fertilization with embryo transfer (IVF-ET) offers a timely opportunity for conception once complete pathological remission is achieved [150].

For patients with Lynch syndrome-associated endometrial cancer (LS-EC), ART is considered a crucial component of management after successful progestin-based therapy. Early initiation of ART, particularly IVF with frozen-thawed embryo transfer (FET), is associated with improved reproductive outcomes [152]. Furthermore, the integration of preimplantation genetic testing for monogenic disorders (PGT-M) within ART cycles for LS-EC patients provides a valuable tool to prevent the transmission of pathogenic mismatch repair gene variants to offspring [152].

Safety remains a paramount concern. Current evidence supports that fertility-preserving approaches, including subsequent ART, do not appear to compromise oncologic survival for well-selected patients [105]. For endometrial cancer patients, ART enables childbearing without necessarily compromising long-term prognosis [153]. However, clinical challenges exist. Repeated endometrial sampling during conservative treatment may lead to a thin endometrium, potentially impairing endometrial receptivity and negatively affecting implantation rates in ART cycles [154]. To mitigate this and other risks, maintenance therapy (e.g. low-dose progestins, levonorgestrel-releasing intrauterine devices) after achieving remission is recommended, as it can protect the endometrium and significantly reduce the risk of recurrence, including after ART procedures [155].

### 5.2. Public health value: highlighting disparities and promoting population health

The application of ART in oncofertility underscores significant health disparities, presenting a critical public health issue. A large, population-based cohort study revealed non-uniform patterns of sociodemographic disparities in the receipt of both fertility-sparing treatment and ART among patients with cervical, endometrial and ovarian cancers [156]. While fertility-sparing surgery was more common among some racial and ethnic minority groups with cervical and ovarian cancers, the use of ART was strongly associated with non-Hispanic White race, younger age (18–35 years), and having private insurance [156]. These findings illuminate inequities in access to advanced reproductive technologies, indicating a public health imperative to address barriers and ensure equitable access for all eligible young cancer patients.

Furthermore, with rising incidence rates of gynaecologic cancers in younger women [157] and improving survival rates [135], a growing population of survivors faces potential infertility. Facilitating access to and standardization of FP and ART services can reduce the disease burden associated with infertility, enhance long-term quality of life, and improve the overall well-being of this population, yielding positive public health benefits.

### 5.3. Policy value: guiding standardized and integrated care

The evidence base calls for policy actions to integrate FP counselling and ART access into standard oncology care pathways through clear guidelines and supportive frameworks.

First, policy should foster and formalize multidisciplinary collaboration (MDT). Multiple reviews emphasize that optimizing reproductive outcomes requires close cooperation between gynaecologic oncologists and reproductive endocrinology/infertility specialists [135]. This collaboration must be initiated promptly at diagnosis to ensure patients are counselled on FP options before commencing potentially gonadotoxic cancer therapy [143].

Second, policy must drive the standardization of clinical practice. A national survey in Spain demonstrated considerable heterogeneity among centres regarding age limits for offering FP, staging criteria, and treatment protocols for different cancers [136]. This variability underscores the need for broader dissemination and adoption of international clinical guidelines to homogenize care. For specific patient subgroups, such as those with LS-EC, policy support is needed for complex services like combining ART with PGT-M, which involves ethical, technical and reimbursement considerations [152].

In summary, ART is an indispensable tool for realizing fertility goals in survivors of reproductive system cancers. Its role extends beyond the clinic, serving as a lens to examine and address health inequities, thereby demanding coordinated policy responses to ensure effective, safe and equitable access for all patients.

## 6. Conclusion

The application of ART in preserving fertility for women diagnosed with malignant tumours of the reproductive system has advanced considerably in recent years. However, critical research gaps persist. The heterogeneity of cancer types, treatment regimens and individual patient responses complicates the establishment of standardized FP protocols. Moreover, the psychological impact of a cancer diagnosis and its treatment on reproductive decision-making warrants significant attention. Future studies should prioritize longitudinal assessments of FP strategies – including ART success rates among cancer survivors – as well as the psychosocial implications of these decisions.

From an expert standpoint, a multidisciplinary approach that integrates insights from oncologists, reproductive endocrinologists and mental health professionals is essential. Such collaboration will foster a more comprehensive understanding of ART’s role in post-cancer reproductive health. Concurrently, continuous education for healthcare providers regarding the latest advancements in FP techniques is crucial. The implementation of standardized, evidence-based guidelines and protocols can optimize FP management for cancer patients, ensuring care is tailored to their individual circumstances.

In summary, while ART has significantly enhanced FP options for women with reproductive system malignancies, substantial gaps remain in terms of accessibility, protocol standardization and the translation of emerging technologies into clinical practice. Addressing these challenges requires sustained research efforts and systematic implementation of multidisciplinary care models.

Key priorities for future research may include: (1) Long-term safety of ovarian tissue transplantation (follow-up ≥10 years) to assess tumour recurrence and offspring health, addressing clinical urgency. This priority targets the most widely used but understudied aspect of clinical practice, directly impacting patient counselling and decision-making. (2) Development of low-cost FP technologies (e.g. point-of-care cryopreservation devices) for LMICs, addressing for global equity). This priority responds to the manuscript’s highlighted global disparities and unmet needs in resource-limited settings. (3) Optimization of artificial ovaries and stem cell-derived gametes to improve clinical translation (e.g. reducing aneuploidy risk), addressing unmet patient needs. This priority focuses on technologies that can benefit patients ineligible for mature FP methods (e.g. prepubertal girls, high-risk malignancies). (4) Establishment of global standardized protocols for ART in cancer patients, including age-specific and tumour-specific guidelines. This priority addresses the manuscript’s highlighted variability in clinical practice and lack of unified guidelines. (5) Ethical and legal frameworks for emerging technologies (e.g. gene editing in FP) to balance innovation and patient safety, addressing future challenges.

## Data Availability

There is no data associated with this research.

## References

[1] Taylan E, Oktay K. Fertility preservation in gynecologic cancers. Gynecol Oncol. 2019;155(3):522–529. doi: 10.1016/j.ygyno.2019.09.012. 31604663

[2] Saenz-de-Juano MD, Ivanova E, Billooye K, et al. Genome-wide assessment of DNA methylation in mouse oocytes. Clin Epigenet. 2019;11(1):197. doi: 10.1186/s13148-019-0794-y. PMC6923880 31856890

[3] Vermeulen M, Giudice MG, Del Vento F, et al. Role of stem cells in fertility preservation. Stem Cells Cloning. 2019;12:27–48. 31496751 10.2147/SCCAA.S178490 PMC6689135

[4] Casciani V, Monseur B, Cimadomo D, et al. Oocyte and embryo cryopreservation in assisted reproductive technology. Fertil Steril. 2023;120(3 Pt):506–520. 37290552 10.1016/j.fertnstert.2023.06.005

[5] Takeda M, Kataoka A, Abe T, et al. Childbirth after perioperative systemic therapy in patients with breast cancer. Jpn J Clin Oncol. 2023;53(6):457–462. 36974683 10.1093/jjco/hyad023

[6] Esfandyari S, Elkafas H, Chugh RM, et al. Exosomes as biomarkers for female reproductive diseases diagnosis and therapy. Int J Mol Sci. 2021;22(4):2165. doi: 10.3390/ijms22042165. 33671587 PMC7926632

[7] Chadchan SB, Singh V, Kommagani R. Female reproductive dysfunctions and the gut microbiota. J Mol Endocrinol. 2022;69(3):R81–R94. doi: 10.1530/JME-21-0238. 35900833 PMC10031513

[8] Popescu-Spineni DM, Guja L, Cristache CM, et al. The influence of endocannabinoid system on women reproduction. Acta Endocrinol (Buchar). 2022;18(2):209–215. doi: 10.4183/aeb.2022.209. 36212249 PMC9512370

[9] Hong IS. Endometrial stem/progenitor cells: properties, origins, and functions. Genes Dis. 2023;10(3):931–947. doi: 10.1016/j.gendis.2022.08.009. 37396532 PMC10308170

[10] Elizondo-Montemayor L, Hernández-Escobar C, Lara-Torre E, et al. Gynecologic and obstetric consequences of obesity in adolescent girls. J Pediatr Adolesc Gynecol. 2017;30(2):156–168. doi: 10.1016/j.jpag.2016.02.007. 26915924

[11] Di Paola R, Costantini C, Tecchio C, et al. Anti-Müllerian hormone and antral follicle count. Oncologist. 2013;18(12):1307–1314. 24149138 10.1634/theoncologist.2013-0138 PMC3868425

[12] Anderson RA, Cameron D, Clatot F, et al. Anti-Müllerian hormone as a marker of ovarian reserve. Hum Reprod Update. 2022;28(3):417–434. 35199161 10.1093/humupd/dmac004 PMC9071067

[13] Tanaka Y, Amano T, Nakamura A, et al. mTOR inhibitors potentially preserve fertility in female patients with haematopoietic malignancies. Ann Hematol. 2024;103(12):4953–4969. doi: 10.1007/s00277-024-06090-3. 39537993

[14] Donovan EK, Covens AL, Kupets RS, et al. The role of oophoropexy in patients with gynecological cancer. Int J Gynecol Cancer. 2022;32(3):380–388. 35256427 10.1136/ijgc-2021-002471

[15] Deligiannis SP, Li T, Moussaud-Lamodière E, et al. Acute high-dose irradiation disrupts cell adhesion. J Ovarian Res. 2026;19(1), 79. doi: 10.1186/s13048-025-01932-8. 41485059 PMC12930946

[16] Habib N, Idoubba S, Futcher F, et al. Cervical cancer treatment and fertility. Cancers (Basel). 2025;17(18):3057. doi: 10.3390/cancers17183057. 41008899 PMC12469036

[17] Jorgensen K, Meernik C, Wu CF, et al. Disparities in fertility-sparing treatment and use of assisted reproductive technology after a diagnosis of cervical, ovarian, or endometrial cancer. Obstet Gynecol. 2023;141(2):341–353. doi: 10.1097/AOG.0000000000005044. 36649345 PMC9858239

[18] Karakaş H, Tarçın G, Bayramoğlu E, et al. Long-term endocrine outcomes in females with acute lymphoblastic leukemia. Ann Hematol. 2026;105(1):7. doi: 10.1007/s00277-026-06783-x. 41530579 PMC12799657

[19] Feichtinger M, Rodriguez-Wallberg KA. Fertility preservation in women with cervical, endometrial or ovarian cancers. Gynecol Oncol Res Pract. 2016;3(1):8. doi: 10.1186/s40661-016-0029-2. 27468354 PMC4962474

[20] Gaughran J, Rosen O’Sullivan H, Lyne T, et al. Fertility preserving surgery outcomes for ovarian malignancy. J Clin Med. 2022;11(11):3195. doi: 10.3390/jcm11113195. 35683582 PMC9181136

[21] Gonçalves V, Ferreira PL, Saleh M, et al. Perspectives of young women with gynecologic cancers on fertility. Oncologist. 2022;27(3):e251–e264. 35274725 10.1093/oncolo/oyab051 PMC8914481

[22] Bayramoglu Z, Timur B, Kızmazoglu D, et al. Pediatric and young adult ovarian masses. Front Pediatr. 2025;13:1639582. doi: 10.3389/fped.2025.1639582. 41000187 PMC12459912

[23] Ertas IE, Taskin S, Goklu R, et al. Long-term oncological and reproductive outcomes of fertility-sparing cytoreductive surgery. J Obstet Gynaecol Res. 2014;40(3):797–805. 24320102 10.1111/jog.12253

[24] Arecco L, de Moura Leite L, Gentile G, et al. Gonadotoxicity of immunotherapy and targeted agents. Hum Reprod. 2025;40(8):1452–1466. 40482082 10.1093/humrep/deaf096 PMC12314150

[25] Christianson MS. Oncofertility and female fertility preservation. Menopause. 2025;32(7):655–657. 40586592 10.1097/GME.0000000000002558

[26] Vesztergom D, Székely B, Hegyi B, et al. Fertility preservation in female cancer patients. Orv Hetil. 2023;164(29):1134–1145. doi: 10.1556/650.2023.32824. 37481767

[27] Robson D, Phua C, Howard R, et al. Fertility preservation in oncology patients. Aust N Z J Obstet Gynaecol. 2020;60(1):18–26. 31617210 10.1111/ajo.13081

[28] Dolmans MM. Recent advances in fertility preservation and counseling for female cancer patients. Expert Rev Anticancer Ther. 2018;18(2):115–120. doi: 10.1080/14737140.2018.1415758. 29220203

[29] Wang Y, Yang JX. Fertility-preserving treatment in women with early endometrial cancer. Cancer Manag Res. 2018;10:6803–6813. 30584372 10.2147/CMAR.S188087 PMC6289121

[30] Sellami I, Saïs E, Souare F, et al. Fertility preservation in women with cervical cancer. Semin Reprod Med. 2025;43(1):5–10. doi: 10.1055/s-0045-1810055. 40749703

[31] Georgescu ES, Goldberg JM, Du Plessis SS, et al. Present and future fertility preservation strategies for female cancer patients. Obstet Gynecol Surv. 2008;63(11):725–732. doi: 10.1097/OGX.0b013e318186aaea. 18928577

[32] Ronn R, Holzer HE. Oncofertility in Canada. Curr Oncol. 2014;21(1):e137–e146. 24523611 10.3747/co.20.1360 PMC3921038

[33] Fisch B, Abir R. Female fertility preservation: past, present and future. Reproduction. 2018;156(1):F11–F27. doi: 10.1530/REP-17-0483. 29581237

[34] Benor A, Decherney A. Gonadotropin-releasing hormone (GnRH) agonists do not protect ovarian function. Cureus. 2024;16(4):e58387. doi: 10.7759/cureus.58387. 38756303 PMC11097921

[35] Jiang YL, Lin YY, Chen CX, et al. Current research of Assisted Reproductive Technology for women with early endometrial cancer and atypical endometrial hyperplasia after conservative treatment. Front Endocrinol (Lausanne). 2024;15:1377396. doi: 10.3389/fendo.2024.1377396. 38919483 PMC11196392

[36] Kalluru S, Vu M, Brady PC. Fertility preservation for cancer. Fertil Steril. 2025;124(4):585–592. 40854469 10.1016/j.fertnstert.2025.08.017

[37] Voigt P, Persily J, Blakemore JK, et al. Sociodemographic differences in utilization of fertility services. J Assist Reprod Genet. 2022;39(4):963–972. 35316438 10.1007/s10815-022-02455-7 PMC9051007

[38] Selter J, Huang Y, Grossman Becht LC, et al. Use of fertility preservation services in female reproductive-aged cancer patients. Am J Obstet Gynecol. 2019;221(4):328.e1–328.e16. doi: 10.1016/j.ajog.2019.05.009. 31108063

[39] Walasik I, Falis M, Płaza O, et al. Polish female cancer survivors’ experiences related to fertility preservation procedures. J Adolesc Young Adult Oncol. 2023;12(5):727–734. doi: 10.1089/jayao.2022.0092. 36719988

[40] Ojo AS, Lipscombe C, Araoye MO, et al. Global uptake of fertility preservation by women undergoing cancer treatment. Cancer Epidemiol. 2022;79:102189. 35605436 10.1016/j.canep.2022.102189

[41] Buchanan Lunsford N, Ragan K, Lee Smith J, et al. Environmental and psychosocial barriers to cervical cancer screening. Oncologist. 2017;22(2):173–181. 28167567 10.1634/theoncologist.2016-0213 PMC5330703

[42] Rodriguez-Wallberg KA, Marklund A, Lundberg F, et al. A prospective study of women and girls undergoing fertility preservation. Acta Obstet Gynecol Scand. 2019;98(5):604–615. 30723910 10.1111/aogs.13559

[43] Lawson AK, McGuire JM, Noncent E, et al. Disparities in counseling female cancer patients for fertility preservation. J Womens Health (Larchmt). 2017;26(8):886–891. doi: 10.1089/jwh.2016.5997. 28498013 PMC5576204

[44] Benedict C, Thom B, Kelvin JF. Young adult female cancer survivors’ decision regret about fertility preservation. J Adolesc Young Adult Oncol. 2015;4(4):213–218. doi: 10.1089/jayao.2015.0002. 26697271 PMC4684663

[45] Wang M, Yang C. Fertility preservation in female children and adolescent cancer patients. Children (Basel). 2025;12(5):647. doi: 10.3390/children12050647. 40426826 PMC12109937

[46] Kim H, Kim H, Ku SY. Fertility preservation in pediatric and young adult female cancer patients. Ann Pediatr Endocrinol Metab. 2018;23(2):70–74. doi: 10.6065/apem.2018.23.2.70. 29969877 PMC6057020

[47] Somigliana E, Mangili G, Martinelli F, et al. Fertility preservation in women with cervical cancer. Crit Rev Oncol Hematol. 2020;154:103092. doi: 10.1016/j.critrevonc.2020.103092. 32896752

[48] Mahmood S, Drakeley A, Homburg R, et al. Fertility preservation in female patients with cancer. Clin Oncol (R Coll Radiol). 2022;34(8):508–513. doi: 10.1016/j.clon.2022.03.021. 35491364

[49] Chu Y, Zhang J, Wang L, et al. The mechanism and protective strategies of follicle injury. J Ovarian Res. 2025;18(1):217. doi: 10.1186/s13048-025-01793-1. 41035050 PMC12487330

[50] Roopnarinesingh R, Tulandi T. Fertility preservation in female cancer patients: surgical procedures. Int J Gynaecol Obstet. 2025;169(3):872–875. doi: 10.1002/ijgo.16173. 39817445 PMC12093914

[51] Erden M, Rehman S, Oh C, et al. Fertility-sparing treatments in patient with gynecologic cancers. Obstet Gynecol. 2026;147(2):186–197. doi: 10.1097/AOG.0000000000006141. 41343835

[52] McQuillan S, Todd N. Clinical consensus statement no. 459: oncofertility. J Obstet Gynaecol Can. 2025;47(3):102807. 40107835 10.1016/j.jogc.2025.102807

[53] Kristensen SG, Pors SE, Poulsen LC, et al. Time from referral to ovarian tissue cryopreservation. Acta Obstet Gynecol Scand. 2019;98(5):616–624. 30758835 10.1111/aogs.13575

[54] Díaz-García C, Herraiz S, Such E, et al. Dexamethasone does not prevent malignant cell reintroduction. Hum Reprod. 2019;34(8):1485–1493. 31339993 10.1093/humrep/dez115

[55] Kasaven LS, Jones BP, Keays R, et al. Anaesthetic considerations for fertility-sparing surgery and uterine transplantation. Anaesthesia. 2021;76 Suppl 4:46–55. doi: 10.1111/anae.15389. 33682092

[56] Caponas G, Deans R, Letafat S, et al. Anaesthesia for uterine transplant and associated surgeries. Int J Obstet Anesth. 2025;65:104800. 41175462 10.1016/j.ijoa.2025.104800

[57] Mottram R, Feltbower RG, Jones G, et al. From storage to survivorship. J Pediatr Adolesc Gynecol. 2025;38(1):26–34. 39197581 10.1016/j.jpag.2024.08.009

[58] Choi YJ, Hong YH, Kim S, et al. The experience of fertility preservation in a single tertiary center in Korea. Front Endocrinol (Lausanne). 2022;13:845051. doi: 10.3389/fendo.2022.845051. 35518927 PMC9062070

[59] Dufour S, Gagné SA, Jackson A, et al. Oocyte cryopreservation outcomes in women with hematological malignancies. J Obstet Gynaecol Can. 2025;47(6):102824. 40157648 10.1016/j.jogc.2025.102824

[60] Tsonis O, Kopeika J. Fertility preservation in patients with gynaecologic malignancy. Eur J Obstet Gynecol Reprod Biol. 2023;290:93–100. 37757729 10.1016/j.ejogrb.2023.09.020

[61] Holtzman S, McCarthy L, Estevez SL, et al. Fertility preservation among hereditary breast and ovarian cancer syndrome previvors. Gynecol Oncol. 2024;186:176–181. 38696905 10.1016/j.ygyno.2024.03.025

[62] Rodgers RJ, Reid GD, Koch J, et al. The safety and efficacy of controlled ovarian hyperstimulation. Hum Reprod. 2017;32(5):1033–1045. 28333356 10.1093/humrep/dex027

[63] Balkenende EM, Dahhan T, Linn SC, et al. Levels of tamoxifen metabolites in controlled ovarian stimulation. Hum Reprod. 2013;28(4):953–959. 23335608 10.1093/humrep/des445

[64] Lifshitz D, Ben-Haroush A, Meirow D, et al. Letrozole or tamoxifen co-administration during fertility preservation. Reprod Biomed Online. 2025;51(3):104878. 40680551 10.1016/j.rbmo.2025.104878

[65] Song BB, Quinn MM. Planned oocyte cryopreservation. Obstet Gynecol Clin North Am. 2023;50(4):707–719. doi: 10.1016/j.ogc.2023.08.005. 37914489

[66] Pai HD, Baid R, Palshetkar NP, et al. Oocyte cryopreservation – current scenario and future perspectives. J Hum Reprod Sci. 2021;14(4):340–349. doi: 10.4103/jhrs.jhrs_173_21. 35197678 PMC8812387

[67] Vervier J, Jouan C, Nisolle M, et al. Oocyte cryopreservation: medical and social indications. Rev Med Liege. 2021;76(9):683–688. 34477340

[68] Mascarenhas M, Mehlawat H, Kirubakaran R, et al. Live birth and perinatal outcomes using cryopreserved oocytes. Hum Reprod. 2021;36(5):1416–1426. 33313698 10.1093/humrep/deaa343

[69] Köroğlu N, Aydın T. Oocyte vitrification for oncological and social reasons. Turk J Obstet Gynecol. 2023;20(1):59–63. doi: 10.4274/tjod.galenos.2022.59827. 36908095 PMC10013077

[70] Lee JA, Sekhon L, Grunfeld L, et al. In-vitro maturation of germinal vesicle and metaphase I eggs. Curr Opin Obstet Gynecol. 2014;26(3):168–173. 24752002 10.1097/GCO.0000000000000062

[71] Lee JA, Barritt J, Moschini RM, et al. Optimizing human oocyte cryopreservation. Fertil Steril. 2013;99(5):1356–1362. 23266213 10.1016/j.fertnstert.2012.11.042

[72] Pereira N, Hubschmann AG, Lekovich JP, et al. Ex vivo retrieval and cryopreservation of oocytes. Fertil Steril. 2017;108(2):357–360. 28629583 10.1016/j.fertnstert.2017.05.025

[73] Whyte JS, Hawkins E, Rausch M, et al. In vivo oocyte retrieval in a young woman with ovarian cancer. Obstet Gynecol. 2014;124(2 Pt 2 Suppl 1):484–486. doi: 10.1097/AOG.0000000000000304. 25004306

[74] Yoshiba T, Takei Y, Manaka Y, et al. A patient with a mucinous borderline ovarian tumor. J Obstet Gynaecol Res. 2022;48(10):2635–2639. 35871537 10.1111/jog.15365 PMC13469777

[75] El Hajj H, Tran PL, Collin-Bund V, et al. Fertility preservation in gynecologic oncology. Int J Gynecol Cancer. 2026;36(1):102782. doi: 10.1016/j.ijgc.2026.104475. 41494211

[76] Lambertini M, Ginsburg ES, Partridge AH. Update on fertility preservation in young women. Curr Opin Obstet Gynecol. 2015;27(1):98–107. 25490381 10.1097/GCO.0000000000000138

[77] Swain M, Miller M, Cannella C, et al. A retrospective study of fertility counseling and preservation rates. Clin Breast Cancer. 2024;24(8):e764–e771. 39327216 10.1016/j.clbc.2024.09.003

[78] Strowitzki T. In vitro maturation (IVM) of human oocytes. Arch Gynecol Obstet. 2013;288(5):971–975. doi: 10.1007/s00404-013-3033-3. 24068296

[79] Gargallo-Alonso M, Picton HM, Malo C. Mechanisms, and clinical perspectives for the in vitro maturation of human oocytes. Int J Mol Sci. 2025;27(1):5. doi: 10.3390/ijms27010005. 41515886 PMC12785462

[80] Grynberg M, El Hachem H, de Bantel A, et al. In vitro maturation of oocytes: uncommon indications. Fertil Steril. 2013;99(5):1182–1188. doi: 10.1016/j.fertnstert.2013.01.090. 23380185

[81] Prasath EB, Chan ML, Wong WH, et al. First pregnancy and live birth resulting from cryopreserved embryos obtained from in vitro matured oocytes after oophorectomy in an ovarian cancer patient. Hum Reprod. 2014;29(2):276–278. doi: 10.1093/humrep/det420.s 24327539

[82] Hatırnaz Ş, Ata B, Hatırnaz ES, et al. Oocyte in vitro maturation: a systematic review. Turk J Obstet Gynecol. 2018;15(2):112–125. doi: 10.4274/tjod.23911. 29971189 PMC6022428

[83] Yee CH, Chung YH, Ko IC, et al. A 6-month sustained-release formulation of triptorelin for locally advanced or metastatic prostate cancer: a real-world experience in Asia. BMC Urol. 2025;25(1):39. doi: 10.1186/s12894-025-01717-7. 40001059 PMC11854001

[84] Heindryckx B, Rybouchkin A, Van Der Elst J, et al. Serial pronuclear transfer increases the developmental potential of in vitro-matured oocytes in mouse cloning. Biol Reprod. 2002;67(6):1790–1795. doi: 10.1095/biolreprod.102.004770. 12444054

[85] Fadini R, Mignini Renzini M, Dal Canto M, et al. Oocyte in vitro maturation in normo-ovulatory women. Fertil Steril. 2013;99(5):1162–1169. doi: 10.1016/j.fertnstert.2013.01.138. 23433517

[86] Morato ALC, Verruma CG, Furtado CLM, et al. In vitro maturation of oocytes: what is already known?†. Biol Reprod. 2025;112(1):18–30. doi: 10.1093/biolre/ioae147. 39423281

[87] Kirillova A, Bunyaeva E, Van Ranst H, et al. Improved maturation competence of ovarian tissue oocytes using a biphasic in vitro maturation system for patients with gynecological malignancy: a study on sibling oocytes. J Assist Reprod Genet. 2021;38(6):1331–1340. doi: 10.1007/s10815-021-02118-z. 33619680 PMC8266929

[88] Yang H, Kolben T, Meister S, et al. Factors influencing the in vitro maturation (IVM) of human oocyte. Biomedicines. 2021;9(12):1904. doi: 10.3390/biomedicines9121904. 34944731 PMC8698296

[89] Li Y, Liu H, Yu Q, et al. Growth hormone promotes in vitro maturation of human oocytes. Front Endocrinol (Lausanne). 2019;10:485. doi: 10.3389/fendo.2019.00485. 31396155 PMC6667636

[90] Seli E, Tangir J. Fertility preservation options for female patients with malignancies. Curr Opin Obstet Gynecol. 2005;17(3):299–308. doi: 10.1097/01.gco.0000169108.15623.34. 15870565

[91] Gunasheela D, Gunasheela S. Strategies for fertility preservation in young patients with cancer: a comprehensive approach. Indian J Surg Oncol. 2014;5(1):17–29. doi: 10.1007/s13193-014-0291-x. 24669162 PMC3964233

[92] Schüring AN, Fehm T, Behringer K, et al. Practical recommendations for fertility preservation in women by the FertiPROTEKT network. Part I: indications for fertility preservation. Arch Gynecol Obstet. 2018;297(1):241–255. doi: 10.1007/s00404-017-4594-3. 29177593 PMC5762797

[93] Cavagna F, Pontes A, Cavagna M, et al. A specific controlled ovarian stimulation (COS) protocol for fertility preservation in women with breast cancer undergoing neoadjuvant chemotherapy. Contemp Oncol (Pozn). 2017;21(4):290–294. doi: 10.5114/wo.2017.72395. 29416435 PMC5799704

[94] Ferreiro E, de Uralde BL, Abreu R, et al. Aromatase inhibitors for ovarian stimulation in patients with breast cancer. Curr Drug Targets. 2020;21(9):910–921. doi: 10.2174/1389450121666200220124607. 32077823

[95] Ní Dhonnabháin B, Elfaki N, Fraser K, et al. A comparison of fertility preservation outcomes in patients who froze oocytes, embryos, or ovarian tissue for medically indicated circumstances: a systematic review and meta-analysis. Fertil Steril. 2022;117(6):1266–1276. doi: 10.1016/j.fertnstert.2022.03.004. 35459522

[96] Chen CY, Yi YC, Guu HF, et al. Pathways to motherhood: a single-center retrospective study on fertility preservation and reproductive outcomes in patients with breast cancer. J Formos Med Assoc. 2025;124(2):112–117. doi: 10.1016/j.jfma.2024.08.005. 39138104

[97] Gu F, Li S, Zheng L, et al. Perinatal outcomes of singletons following vitrification versus slow-freezing of embryos: a multicenter cohort study using propensity score analysis. Hum Reprod. 2019;34(9):1788–1798. doi: 10.1093/humrep/dez095. 31407797

[98] Zhang H, Ye D, Wu Y, et al. Effect of exposed-to-air frequency of cryopreserved embryo on clinical outcomes of vitrified-warmed embryo transfer cycles: a retrospective analysis of 9,200 vitrified-warmed transfer cycles. BMC Pregnancy Childbirth. 2023;23(1):590. doi: 10.1186/s12884-023-05879-w. 37592241 PMC10433674

[99] Porcu E, Cillo GM, Cipriani L, et al. Impact of BRCA1 and BRCA2 mutations on ovarian reserve and fertility preservation outcomes in young women with breast cancer. J Assist Reprod Genet. 2020;37(3):709–715. doi: 10.1007/s10815-019-01658-9. 31872386 PMC7125060

[100] Bahroudi Z, Zarnaghi MR, Izadpanah M, et al. Review of ovarian tissue cryopreservation techniques for fertility preservation. J Gynecol Obstet Hum Reprod. 2022;51(2):102290. doi: 10.1016/j.jogoh.2021.102290. 34906692

[101] Seracchioli R, Maletta M, Pazzaglia E, et al. Ovarian tissue biopsy for cryopreservation by vaginal natural orifice transluminal endoscopic surgery: a new approach for a minimal invasive ovarian biopsy. Fertil Steril. 2024;122(2):385–387. doi: 10.1016/j.fertnstert.2024.04.005. 38604263

[102] Poulain M, Vandame J, Tran C, et al. Fertility preservation in borderline ovarian tumor patients and survivors. Horm Mol Biol Clin Investig. 2020;43(2):179–186. doi: 10.1515/hmbci-2019-0072. 32628631

[103] Santos ML, Pais AS, Almeida Santos T. Fertility preservation in ovarian cancer patients. Gynecol Endocrinol. 2021;37(6):483–489. doi: 10.1080/09513590.2021.1872534. 33501866

[104] Kristensen SG, Giorgione V, Humaidan P, et al. Fertility preservation and refreezing of transplanted ovarian tissue – a potential new way of managing patients with low risk of malignant cell recurrence. Fertil Steril. 2017;107(5):1206–1213. doi: 10.1016/j.fertnstert.2017.03.017. 28433369

[105] Vitale F, Dolmans MM. Comprehensive review of in vitro human follicle development for fertility restoration: recent achievements, current challenges, and future optimization strategies. J Clin Med. 2024;13(6):1791. doi: 10.3390/jcm13061791. 38542015 PMC10970962

[106] Geoffron S, Lier A, de Kermadec E, et al. Fertility preservation in women with malignant and borderline ovarian tumors: experience of the French ESGO-certified center and pregnancy-associated cancer network (CALG). Gynecol Oncol. 2021;161(3):817–824. doi: 10.1016/j.ygyno.2021.03.030. 33812696

[107] Pors SE, Ramløse M, Nikiforov D, et al. Initial steps in reconstruction of the human ovary: survival of pre-antral stage follicles in a decellularized human ovarian scaffold. Hum Reprod. 2019;34(8):1523–1535. doi: 10.1093/humrep/dez077. 31286144

[108] Mutlu L, Manavella DD, Gullo G, et al. Endometrial cancer in reproductive age: fertility-sparing approach and reproductive outcomes. Cancers (Basel). 2022;14(21):5187. doi: 10.3390/cancers14215187. 36358604 PMC9656291

[109] Akel RA, Guo XM, Moravek MB, et al. Ovarian stimulation is safe and effective for patients with gynecologic cancer. J Adolesc Young Adult Oncol. 2020;9(3):367–374. doi: 10.1089/jayao.2019.0124. 31923372 PMC7307696

[110] Polyzos NP, Mauri D, Tsioras S, et al. Intraperitoneal dissemination of endometrial cancer cells after hysteroscopy: a systematic review and meta-analysis. Int J Gynecol Cancer. 2010;20(2):261–267. doi: 10.1111/igc.0b013e3181ca2290. 20169669

[111] Chang YN, Zhang Y, Wang YJ, et al. Effect of hysteroscopy on the peritoneal dissemination of endometrial cancer cells: a meta-analysis. Fertil Steril. 2011;96(4):957–961. doi: 10.1016/j.fertnstert.2011.07.1146. 21872230

[112] Giampaolino P, Cafasso V, Boccia D, et al. Fertility-sparing approach in patients with endometrioid endometrial cancer grade 2 stage IA (FIGO): a qualitative systematic review. Biomed Res Int. 2022;2022:4070368. doi: 10.1155/2022/4070368. 36203482 PMC9532104

[113] Paweena T, Leonel ECR, Fernando RRT, et al. Human ovarian tissue xenotransplantation: advancements, challenges, and future perspectives. Hum Reprod. 2025;40(3):410–419. 39749868 10.1093/humrep/deae291

[114] Karimizadeh Z, Saltanatpour Z, Tarafdari A, et al. Ovarian tissue cryopreservation: a narrative review on cryopreservation and transplantation techniques, and the clinical outcomes. Ther Adv Reprod Health. 2025;19:26334941251340517. doi: 10.1177/26334941251340517. 40463479 PMC12130658

[115] Chen J, Cao D, Yang J, et al. Fertility-sparing treatment for endometrial cancer or atypical endometrial hyperplasia patients with obesity. Front Oncol. 2022;12:812346. doi: 10.3389/fonc.2022.812346. 35251982 PMC8895268

[116] Chen J, Cao D. Fertility-sparing re-treatment for endometrial cancer and atypical endometrial hyperplasia patients with progestin-resistance: a retrospective analysis of 61 cases. World J Surg Oncol. 2024;22(1):169. doi: 10.1186/s12957-024-03439-w. 38918837 PMC11202344

[117] Pino I, Iacobone AD, Vidal Urbinati AM, et al. Fertility-sparing treatment for endometrial cancer: oncological and obstetric outcomes in combined therapies with levonorgestrel intrauterine device. Cancers (Basel). 2022;14(9):2170. doi: 10.3390/cancers14092170. 35565299 PMC9101107

[118] De Rocco S, Buca D, Oronzii L, et al. Reproductive and pregnancy outcomes of fertility-sparing treatments for early-stage endometrial cancer or atypical hyperplasia: a systematic review and meta-analysis. Eur J Obstet Gynecol Reprod Biol. 2022;273:90–97. doi: 10.1016/j.ejogrb.2022.04.019. 35526471

[119] Wang Y, Bo L, Fan X, et al. Molecular classification guides fertility-sparing treatment for endometrial cancer and atypical hyperplasia patients. Curr Oncol. 2025;32(6):317. doi: 10.3390/curroncol32060317. 40558260 PMC12192213

[120] Donnez J, Dolmans MM. Fertility preservation in women. N Engl J Med. 2017;377(17):1657–1665. doi: 10.1056/NEJMra1614676. 29069558

[121] Telfer EE, Zelinski MB. Ovarian follicle culture: advances and challenges for human and nonhuman primates. Fertil Steril. 2013;99(6):1523–1533. doi: 10.1016/j.fertnstert.2013.03.043. 23635350 PMC3929501

[122] White YAR, Woods DC, Takai Y, et al. Oocyte formation by mitotically active germ cells purified from ovaries of reproductive-age women. Nat Med. 2012;18(3):413–421. doi: 10.1038/nm.2669. 22366948 PMC3296965

[123] Lambertini M, Peccatori FA, Demeestere I, et al. Fertility preservation and post-treatment pregnancies in post-pubertal cancer patients: ESMO Clinical Practice Guidelines. Ann Oncol. 2020;31(12):1664–1678. doi: 10.1016/j.annonc.2020.09.006. 32976936

[124] Bedoschi G, Navarro PA, Oktay K. Chemotherapy-induced damage to ovary: mechanisms and clinical impact. Future Oncol. 2016;12(20):2333–2344. doi: 10.2217/fon-2016-0176. 27402553 PMC5066134

[125] Woodruff TK, et al. The Oncofertility Consortium – addressing fertility in young cancer patients. Nat Rev Clin Oncol. 2010;7(8):466–475. 20498666 10.1038/nrclinonc.2010.81 PMC3124936

[126] Gellert SE, Pors SE, Kristensen SG, et al. Long-term follow-up of children born after autologous grafting of cryopreserved ovarian tissue. Hum Reprod. 2022;37(5):1026–1035.

[127] Marin L, Vural NA, Oktay KH. Advances to fertility preservation in patients with gynecological cancers. Expert Rev Endocrinol Metab. 2026;21(1):43–52. doi: 10.1080/17446651.2025.2596680. 41327914

[128] Pajot E, Muñoz Sastre MT, Mullet E. Mapping French people’s views regarding posthumous reproduction. J Reprod Infant Psychol. 2017;35(5):524–537. doi: 10.1080/02646838.2017.1371283. 29517382

[129] Huang J, Li J, Xiao W, et al. Attitudes toward posthumous assisted reproduction in China: a multi-dimensional survey. Reprod Health. 2022;19(1):122. doi: 10.1186/s12978-022-01423-9. 35598020 PMC9124412

[130] Piergentili R, Del Rio A, Signore F, et al. CRISPR-Cas and its wide-ranging applications: from human genome editing to environmental implications, technical limitations, hazards and bioethical issues. Cells. 2021;10(5):969. doi: 10.3390/cells10050969. 33919194 PMC8143109

[131] Rumpik D, Rumpikova T, Pohanka M, et al. Gestational surrogacy in the Czech Republic. Biomed Pap Med Fac Univ Palacky Olomouc Czech Repub. 2019;163(2):155–160. doi: 10.5507/bp.2018.040. 30238935

[132] Yu RS, Yu HC, Yang YF, et al. A global overview of per- and polyfluoroalkyl substance regulatory strategies and their environmental impact. Toxics. 2025;13(4):251. doi: 10.3390/toxics13040251. 40278567 PMC12030800

[133] Espenel S, Pointreau Y, Genestie C[, et al. Molecular-integrated risk profile: an opportunity for therapeutic de-escalation in intermediate and high-intermediate risk endometrial cancer]. Cancer Radiother. 2022;26(6-7):931–937. doi: 10.1016/j.canrad.2022.06.007. 36031498

[134] Mitric C, Bernardini MQ. Endometrial cancer: transitioning from histology to genomics. Curr Oncol. 2022;29(2):741–757. doi: 10.3390/curroncol29020063. 35200562 PMC8870297

[135] Gullo G, Scaglione M, Buzzaccarini G, et al. Cell-free fetal DNA and non-invasive prenatal diagnosis of chromosomopathies and pediatric monogenic diseases: a critical appraisal and medicolegal remarks. J Pers Med. 2022;13(1):1. doi: 10.3390/jpm13010001. 36675662 PMC9862851

[136] Panay N, Anderson RA, Bennie A, ESHRE, ASRM, CREWHIRL, and IMS Guideline Group on POI., et al. Evidence-based guideline: premature ovarian insufficiency. Hum Reprod Open. 2024;2024(4):hoae065. doi: 10.1093/hropen/hoae065. 39660328 PMC11631070

[137] Muscaritoli M, Molfino A, Orlando S, et al. Advancements of investigational agents for cancer cachexia: what clinical progress have we seen in the last 5 years? Expert Opin Investig Drugs. 2025;34(11):855–867. doi: 10.1080/13543784.2025.2588640. 41222020

[138] Ohara T, Kuji S, Takenaga T, et al. Current state of fertility preservation for adolescent and young adult patients with gynecological cancer. Int J Clin Oncol. 2022;27(1):25–34. doi: 10.1007/s10147-021-02063-y. 34779961

[139] Gorostidi M, Gil-Ibañez B, Alonso S, et al. Fertility preservation treatment of gynecological cancer patients in Spain: a national survey (GOFER study). Arch Gynecol Obstet. 2020;301(3):793–800. doi: 10.1007/s00404-020-05468-8. 32124016

[140] Plante M, Renaud MC, Sebastianelli A, et al. Simple vaginal trachelectomy in women with early-stage low-risk cervical cancer who wish to preserve fertility: the new standard of care? Int J Gynecol Cancer. 2020;30(7):981–986. doi: 10.1136/ijgc-2020-001432. 32499393

[141] Moro F, Bonanno GM, Gui B, et al. Imaging modalities in fertility preservation in patients with gynecologic cancers. Int J Gynecol Cancer. 2021;31(3):323–331. doi: 10.1136/ijgc-2020-002109. 33139315

[142] Knez J, Al Mahdawi L, Takač I, et al. The perspectives of fertility preservation in women with endometrial cancer. Cancers (Basel). 2021;13(4):602. doi: 10.3390/cancers13040602. 33546293 PMC7913307

[143] Ameri A, Novin K, Sourati A, et al. Awareness of female cancer patients about the risk of impaired fertility. J Adolesc Young Adult Oncol. 2019;8(3):342–348. doi: 10.1089/jayao.2018.0112. 30585751

[144] Cakmak H, Katz A, Cedars MI, et al. Effective method for emergency fertility preservation: random-start controlled ovarian stimulation. Fertil Steril. 2013;100(6):1673–1680. doi: 10.1016/j.fertnstert.2013.07.1992. 23987516

[145] Floyd JL, Campbell S, Rauh-Hain JA, et al. Fertility preservation in women with early-stage gynecologic cancer: optimizing oncologic and reproductive outcomes. Int J Gynecol Cancer. 2021;31(3):345–351. doi: 10.1136/ijgc-2020-001328. 32565487 PMC12038822

[146] Zaami S, Melcarne R, Patrone R, et al. Oncofertility and reproductive counseling in patients with breast cancer: a retrospective study. J Clin Med. 2022;11(5):1311. doi: 10.3390/jcm11051311. 35268402 PMC8911138

[147] Herrera Cappelletti E, Humann J, Torrejón R, et al. Chances of pregnancy and live birth among women undergoing conservative management of early-stage endometrial cancer: a systematic review and meta-analysis. Hum Reprod Update. 2022;28(2):282–295. doi: 10.1093/humupd/dmab041. 34935045 PMC8888991

[148] Iliadis SI, Gambadauro P. Conservative management of early-stage endometrial cancer for fertility preservation: a survey study among Swedish gynecologists and gynecological oncologists. Sci Rep. 2023;13(1):5861. doi: 10.1038/s41598-023-32911-y. 37041242 PMC10090158

[149] Wu Y, Wang J, Fan Y, et al. Metabolic syndrome combined with insulin resistance showed great predictive value in evaluating recurrence in patients with atypical endometrial hyperplasia and early endometrial cancer. BMC Cancer. 2025;25(1):1094. doi: 10.1186/s12885-025-14481-6. 40597086 PMC12210504

[150] Ye R, Mao YM, Fei YR, et al. Targeting ferroptosis for the treatment of female reproductive system disorders. J Mol Med (Berl). 2025;103(4):381–402. doi: 10.1007/s00109-025-02528-x. 40100417

[151] Heras M, Gracia M, Coronado P. Robotic radical trachelectomy in early stage cervical cancer. J Robot Surg. 2025;19(1):361. doi: 10.1007/s11701-025-02540-w. 40624221

[152] Tong XM, Lin XN, Jiang HF, et al. Fertility-preserving treatment and pregnancy outcomes in the early stage of endometrial carcinoma. Chin Med J (Engl). 2013;126(15):2965–2971. 23924476

[153] Wang Y, Zhou R, Wang H, et al. Impact of treatment duration in fertility-preserving management of endometrial cancer or atypical endometrial hyperplasia. Int J Gynecol Cancer. 2019;29(4):699–704. doi: 10.1136/ijgc-2018-000081. 30826750

[154] Liu J, Zheng Y, Liu J. Fertility preservation and assisted reproductive strategies in endometrial cancer patients with lynch syndrome. Front Oncol. 2025;15:1630301. doi: 10.3389/fonc.2025.1630301. 40761251 PMC12318751

[155] Carneiro MM, Lamaita RM, Ferreira MCF, et al. Fertility-preservation in endometrial cancer: is it safe? Review of the literature. JBRA Assist Reprod. 2016;20(4):232–239. doi: 10.5935/1518-0557.20160045. 28050959 PMC5265623

[156] Fujimoto A, Ichinose M, Harada M, et al. The outcome of infertility treatment in patients undergoing assisted reproductive technology after conservative therapy for endometrial cancer. J Assist Reprod Genet. 2014;31(9):1189–1194. doi: 10.1007/s10815-014-0297-x. 25106937 PMC4156956

[157] He Y, Wang J, Wang Y, et al. Maintenance therapy can improve the oncologic prognosis and obstetrical outcome of patients with atypical endometrial hyperplasia and endometrial cancer after fertility-preserving treatment: a multicenter retrospective study. Front Oncol. 2021;11:808881. doi: 10.3389/fonc.2021.808881. 34976844 PMC8718436

